# Affinal parenthood after divorce: a socio-legal study of stepparent visitation rights in Peruvian blended families

**DOI:** 10.3389/fsoc.2026.1870230

**Published:** 2026-09-09

**Authors:** José Luis Castilla-Cabezudo, Reimy Zegarra-Castañeda, Giany Zegarra-Castañeda, Claudia Catherine Díaz-Arias, César Jesús Martín Salcedo-Tenemas

**Affiliations:** Facultad de Derecho, Universidad Tecnológica del Perú, Ica, Peru

**Keywords:** affinal parenthood, best interests of the child, blended families, functional parenthood, judicial discretion, Peru, social parenthood, socio-affective kinship

## Abstract

**Introduction:**

Blended families challenge legal systems that continue to organize parental status primarily through formal categories such as filiation, adoption, parental authority, custody, and legally recognized family status. This study examines how Peruvian judicial, legal, prosecutorial, public-defense, and psychosocial professionals interpret the possibility of granting post-divorce visitation rights to stepparents in blended families. It does not propose automatic legal parenthood for stepparents but analyzes whether affinal status, when accompanied by evidence of social or functional parenthood, may provide a basis for judicial assessment under the best interests of the child.

**Methods:**

An exploratory qualitative socio-legal design was employed, combining analysis of Peruvian constitutional, civil, and child-protection law with eleven structured expert interviews. Participants included two family judges, four lawyers, two public defenders, one family prosecutor, and two psychologists in Ica, Peru. Data were analyzed through directed thematic analysis using deductive and inductive coding, coder comparison, and analytic triangulation across interviews, legal materials, jurisprudence, doctrine, and socio-legal literature.

**Results:**

Six interrelated themes were identified: formal affinity as a procedural gateway rather than a complete legal basis; the best interests of the child as a structured judicial inquiry; socio-affective bonds as an evidentiary threshold; non-displacement of biological or legal parents; the relevance of interdisciplinary and psychosocial evidence; and uncertainty arising from judicial discretion. Participants generally considered affinity insufficient by itself and emphasized evidence of caregiving, emotional continuity, and the child’s welfare.

**Discussion:**

The findings indicate that Peruvian law provides interpretive mechanisms for assessing post-divorce stepparent visitation despite the absence of a specific statutory regime. Affinal status may make a claim legally cognizable, but continued contact should depend on evidence that the relationship acquired social or functional parental significance and that visitation serves the child’s best interests without displacing the rights and duties of legal parents. The study contributes a Latin American perspective to broader socio-legal debates on social parenthood, functional parenthood, socio-affective kinship, family diversity, and judicial discretion.

## 1 Introduction

Parenthood is increasingly difficult to understand only through formal legal categories such as biological filiation, adoption, parental authority and custody (Lind, 2024; Lima, 2019). In blended families, the spouse or partner of the child’s legal parent may participate in everyday caregiving, emotional support, educational routines, discipline, economic contribution and family integration without becoming a legal parent (Mason et al., 2002; Malia, 2005). When the adult couple separates, the law faces a specific socio-legal question: whether continued contact between a child and a former stepparent should be subject to judicial assessment when that adult has become a meaningful figure in the child’s lived family world.

This question is not reducible to the adult claimant’s interest in maintaining contact. It concerns the institutional recognition of relationships that may be socially meaningful for the child but only partially named by statute. A former stepparent may have been no more than the spouse of the child’s parent. In other cases, however, the adult may have performed stable caregiving functions, cohabited with the child, contributed to routines of care and been recognized by the child as a parental or quasi-parental figure (Sanner et al., 2022; Tanskanen and Danielsbacka, 2022). The central difficulty is therefore not whether every stepparent should obtain post-divorce rights, but whether courts should be able to assess the continuity of a particular child-adult relationship when the evidence shows a significant socio-affective bond.

The Peruvian case provides a useful setting for examining this problem. Peruvian constitutional discourse protects the family and constitutional jurisprudence has recognized the existence of blended or reconstituted families (Meza et al., 2019; Villa and Hurtado, 2022). Nevertheless, Peruvian civil and child-protection law does not provide a specific and comprehensive regime governing the post-divorce rights and duties of stepparents. The Civil Code recognizes affinity as a legally relevant relationship, and Article 237 provides a legal and doctrinal basis for discussing the persistence of affinity after dissolution of marriage. Article 90 of the Code of Children and Adolescents allows visitation to extend beyond parents to relatives by affinity and, where the child’s best interests require it, to third parties (Bazán, 2022; Shinno, 2023; Varsi, 2020). These provisions create interpretive possibilities, but they do not amount to a clear legislative regime for affinal parenthood after divorce Table 1.

**Table 1:** Analytical model connecting theory, method and results.

Dimension	Question	Evidence	Socio-legal significance
Law in books	What legal categories are available?	Constitution, Civil Code, Code of Children and Adolescents, jurisprudence	Shows the formal legal structure and its limits.
Law in action	How do actors interpret those categories?	Interview data and professional reasoning	Shows how legal meaning is produced in practice.
Lived family	Was there a meaningful caregiving bond?	Cohabitation, care routines, attachment, child identity	Distinguishes formal affinity from functional/social parenthood.
Child contact	Would continued contact serve the child?	Wellbeing, stability, risk, child voice	Frames visitation as child-centered rather than adult entitlement.
Judicial discretion	What risks arise from statutory silence?	Divergent interpretations and lack of criteria	Identifies need for structured criteria or reform.

Peru is not distinctive because it invokes the best interests of the child. That principle is widely present in contemporary family law and child-rights discourse (Archard and Skivenes, 2009; Herencia, 2021; Parker, 1994). Its relevance here lies in a more specific combination: constitutional recognition of blended families, statutory silence regarding post-divorce stepparenthood and dispersed legal categories such as affinity and third-party visitation. This combination makes Peru a useful case for examining how legal and psychosocial actors reason when a child’s lived family relationship is socially significant but only partially named by the statute.

Existing Peruvian scholarship has mainly approached the matter from doctrinal and constitutional perspectives. It has emphasized the lack of express regulation of blended families, the need to protect the child’s best interests and the possibility of grounding stepparent visitation in the interaction between affinity, constitutional family protection and child-rights principles (Bazán, 2022; Meza et al., 2019; Shinno, 2023; Villa and Hurtado, 2022). This doctrinal work is essential because it identifies the legal gap. However, it does not fully explain how that gap is interpreted in practice by legal, judicial, public-defense, prosecutorial and psychosocial professionals.

International debates reinforce the relevance of this inquiry. Socio-legal and family-law scholarship has shown that stepfamilies are socially recurrent but incompletely institutionalized, and that legal systems increasingly face claims involving social parenthood, functional parenthood, psychological parenthood and de facto caregiving (Cherlin, 1978; Huntington et al., 2023; Mason et al., 2002; Raley and Sweeney, 2020; Stewart and Timothy, 2020). Comparative literature does not support a simple opposition between legal form and parental function. Many systems still organize parenthood through formal categories, but several jurisdictions have developed functional or fact-sensitive mechanisms that recognize caregiving, cohabitation, parental responsibility or contact in specific circumstances (Harding and Pawliczak, 2025; Lima, 2019; Scherpe, 2005). The Peruvian case should therefore be situated within this broader trend rather than portrayed as an isolated exception.

This article asks: how do Peruvian judicial, legal, public-defense, prosecutorial and psychosocial professionals interpret the possibility of granting visitation rights to stepparents after divorce in blended families, and what socio-legal role does the best interests principle play in that interpretation? To address this question, the study combines socio-legal analysis of Peruvian constitutional, civil and child-protection materials with structured expert interviews conducted with eleven participants in Ica, Peru: two family judges, four lawyers, two public defenders, one family prosecutor and two psychologists.

The article argues that affinal status can provide an initial legal gateway for a stepparent visitation claim, but it should not automatically justify visitation. The decisive issue is whether the relationship between the child and the stepparent acquired social or functional parental significance and whether continued contact serves the child’s best interests without displacing the rights and duties of biological or legal parents. The contribution is threefold. First, the article reframes Peruvian debates on stepparent visitation as a socio-legal problem of recognition. Second, it shows how legal and psychosocial professionals reason about open legal standards when statutory regulation is incomplete. Third, it provides a Latin American case for comparative discussions on social parenthood, functional parenthood, socio-affective kinship and judicial discretion in family law.

## 2 Theoretical framework

This section develops the conceptual and socio-legal framework used in the empirical analysis. It first explains the distinction between formal legal categories and lived family relationships. It then defines the key concepts used in the article: legal parenthood, affinal parenthood, social parenthood, functional parenthood, de facto caregiving and socio-affective kinship. The section also explains why the best interests principle must be treated as a structured judicial inquiry rather than as a general rhetorical formula.

### 2.1 Family transformation, law and socio-legal recognition

Modern family law continues to rely on formal categories that provide institutional stability: marriage, filiation, adoption, parental authority, custody, guardianship and legally regulated contact. These categories are indispensable because they allocate rights, duties, decision-making authority and legal responsibility. Yet they do not always describe how family relationships are actually lived. Contemporary family arrangements may be organized through post-divorce caregiving, recomposed households, shared parenting across households, kin networks and adult-child relationships based on social rather than biological parenthood (Amato, 2010; Brown and Wade, 2023; Sweeney, 2010).

This article approaches that tension through the classic socio-legal distinction between law in books and law in action (Pound, 1910). Written law is not irrelevant; statutory categories shape the field of possible claims. However, socio-legal analysis asks how law is interpreted, mobilized and adapted when it enters institutional practice. In disputes involving blended families, the written law may provide rules on affinity, visitation and child protection, but the practical meaning of those rules depends on how judges, lawyers, prosecutors, public defenders and psychosocial professionals construct the relevance of care, attachment and family continuity (Felstiner et al., 1981; Johansen et al., 2025).

Socio-legal recognition differs from the mere existence of a statutory provision. Recognition involves the institutional capacity to make a social relationship legally intelligible. A caregiving relationship may be real and emotionally significant, but unless legal actors can translate it into a recognized category—such as affinity, third-party contact, social parenthood or the child’s best interests—it may remain legally fragile (Felstiner et al., 1981; Lind, 2024). This is why the distinction between the legal family and the lived family matters. The legal family is organized through formal statuses; the lived family is organized through practices of belonging, dependency, routine, affection, responsibility and recognition.

Judges occupy a particularly important position when legislation does not expressly regulate the relationship. They do not create the socio-affective bond, but they may determine whether an existing bond will receive legal protection after the adult couple separates. Their role is interpretive because they must apply open legal norms to concrete family histories. It is also institutionally constitutive because their decisions can transform a social relationship into one that produces enforceable contact. This is precisely where a sociology of law approach adds value to doctrinal analysis.

### 2.2 Conceptual distinctions: legal parenthood, affinal parenthood, social parenthood, functional parenthood and de facto caregiving

The article distinguishes concepts that are sometimes used interchangeably but refer to different legal and socio-legal positions (Huntington et al., 2023; Lima, 2025). Legal parenthood refers to a parent–child status formally recognized by law, usually through filiation, adoption or another legally established mechanism that allocates parental authority, duties, decision-making powers and legal responsibility. Legal parenthood is therefore a status with institutional effects. It cannot be inferred merely from affection or participation in a child’s life.

Affinal parenthood refers to the position of the spouse or former spouse of the child’s legal or biological parent, where the adult’s connection to the child derives initially from affinity rather than filiation or adoption. In this article, affinal parenthood is not limited to a specific gender configuration of the adult couple. What matters is whether the adult acquired an affinal position in relation to the child and whether, beyond that formal position, the child experienced the adult as a meaningful caregiving figure. This clarification is important because the focus is not the sex or marital configuration of the adults, but the relationship between the adult claimant and the child (Bazán, 2022; Varsi, 2020).

Social parenthood refers to an adult-child relationship in which the adult performs parental functions in everyday life without necessarily being a legal parent. Functional parenthood emphasizes what the adult actually does: caregiving, emotional support, educational involvement, discipline, protection, economic contribution and participation in the child’s routines. De facto caregiving refers to the practical exercise of care without formal legal status. Socio-affective kinship refers to the relational bond produced by cohabitation, care, attachment, recognition and shared family life (Malia, 2005; Huntington et al., 2023; Mason et al., 2002; Sanner et al., 2022).

These distinctions are central to the argument. A stepparent may be an affinal relative without being a social or functional parent. Conversely, an adult may perform meaningful caregiving functions without acquiring full legal parenthood. The legal issue examined in this article is therefore not whether stepparents should automatically become legal parents after divorce. The issue is whether a former stepparent who has acted as a social or functional parent should have standing to request visitation when continued contact is consistent with the child’s best interests.

The comparative literature on social parenthood supports this differentiated approach. Social Parenthood in Comparative Perspective shows that the term social parenthood must be understood broadly because lived parental relationships arise in diverse jurisdictions and family forms (Huntington et al., 2023). Lima’s work on parenthood in European human-rights jurisprudence also shows that parenthood is not always treated as a purely biological or formal category (Lima, 2019). Scherpe’s work on cohabitants similarly indicates that some legal systems use factual requirements such as cohabitation, duration and relational function as thresholds for legal recognition (Scherpe, 2005). These comparative materials reinforce the need to avoid an overly formalistic account of stepparenthood.

### 2.3 Blended families, stepparenthood and socio-affective kinship

Blended families are usually defined as family arrangements in which one or both members of a couple bring children from previous relationships into a new household or family project (Cherlin, 1978; Coleman et al., 2000; Sweeney, 2010). For socio-legal purposes, however, the importance of blended families lies in their capacity to generate relationships of care that are neither purely biological nor fully formalized as legal parenthood. In these families, a stepparent may take part in daily supervision, school routines, emotional support, discipline, economic contribution and family decision-making. Over time, this role can become part of the child’s relational world and identity (Sanner et al., 2022).

The question in the present study is not whether all stepparents should acquire parental authority. Automatic recognition would risk displacing or interfering with the rights and responsibilities of biological or legal parents. The more precise question is whether, after divorce or separation, a stepparent who has developed a meaningful socio-affective bond with a child should have standing to request child contact, and whether a court may grant visitation when such contact serves the child’s best interests.

International literature supports this cautious approach. Stepfamilies remain incompletely institutionalized: they are socially common but often insufficiently addressed by law and public policy (Cherlin, 1978; Stewart and Timothy, 2020). Comparative work on social parenthood and family diversity also indicates that legal systems increasingly face pressure to accommodate caregiving relationships without converting every caregiver into a full legal parent (Harding and Pawliczak, 2025; Huntington et al., 2023; Lind, 2024). The challenge is not simply to expand parental rights, but to design child-centered responses capable of preserving meaningful relationships while respecting the rights and responsibilities of legal parents.

For that reason, the Peruvian language of padre afín, madre afín or hijo afín should not be treated as a complete solution. The decisive issue is not the label itself but the nature of the relationship that the label may or may not protect. A stepparent who merely existed as a spouse of the child’s parent is not in the same position as a stepparent who provided care, cohabited with the child and was recognized by the child as a parental figure. Any legal recognition of affinal parenthood should therefore be evidence-sensitive, child-centered and limited to the protection of meaningful contact where such contact supports the child’s wellbeing.

### 2.4 The best interests of the child as a structured legal inquiry

The best interests principle is central to the proposed socio-legal framework, but it must be treated carefully. It is not a formula that automatically validates any claim made in the name of child welfare. Nor is it a rhetorical substitute for legal reasoning. It is an open legal standard that directs decision-makers toward the child’s welfare, development, identity, safety and relational needs. Because it is open, it requires contextual interpretation and justification in each case (Archard and Skivenes, 2009; Mnookin, 1975; Parker, 1994).

In this article, the best interests principle is treated as a structured judicial inquiry. In stepparent visitation disputes, courts should examine whether continued contact preserves emotional stability, family identity and relational continuity. They should also examine risk: loyalty conflicts, adult manipulation, violence, coercion, child rejection, weak or superficial bonds and interference with legal parents. The principle therefore works as both a gateway and a limit. It may permit evaluation of a relationship that the statute does not regulate expressly, but it also prevents contact from being granted merely because an adult requests it.

The standard’s strength is flexibility. Family life is complex, and rigid rules may fail to protect children whose relationships do not fit conventional legal categories. However, its risk is indeterminacy. If no specific criteria exist for assessing affinal or socio-affective bonds, similar cases may receive different treatment. One judge may view the former stepparent as a meaningful social parent; another may treat that adult as a third party whose claim should remain exceptional. The same principle can therefore produce protective flexibility or unpredictable variation (Mnookin, 1975; Parker, 1994; Herencia, 2021).

A structured best-interests inquiry should include at least the following elements: duration and intensity of cohabitation; caregiving functions performed by the stepparent; the child’s age and capacity to express views; the child’s perception of the relationship; the position of biological or legal parents; risks of conflict or harm; prior violence or coercion; and the likely effect of continuing or interrupting contact. These criteria help convert an open standard into reasoned adjudication.

### 2.5 The Peruvian legal field: statutory silence, affinity, and judicial discretion

The Peruvian legal field contains elements that can support the recognition of stepparent visitation, but these elements remain dispersed. The Constitution protects the family and provides a framework for child-centered judicial interpretation. Constitutional jurisprudence has recognized that blended families should not be treated as constitutionally irrelevant. Peruvian scholarship has also argued that the exclusion or differential treatment of stepchildren may weaken constitutional family protection (Meza et al., 2019; Shinno, 2023; Villa and Hurtado, 2022). These materials create an interpretive environment in which the family is not limited to the traditional nuclear model.

At the statutory level, two provisions are especially relevant. Article 237 of the Civil Code provides the legal and doctrinal basis for discussing affinity and its persistence after dissolution of marriage. Article 90 of the Code of Children and Adolescents allows visitation rights to be extended beyond parents to certain relatives, including relatives by affinity, and to third parties when the child’s best interests justify it. Read together, these provisions make it possible to argue that a stepparent may request visitation after divorce when the relationship with the child has become socio-affectively significant (Bazán, 2022; Herencia, 2021; Varsi, 2020).

However, these provisions do not expressly define the rights and duties of the padre afín or madre afín, nor do they establish criteria for evaluating the child’s relationship with a stepparent after the adult couple separates. This is the central statutory silence. Peruvian law recognizes affinity, protects children and acknowledges family diversity at a constitutional level, but it does not provide a specific post-divorce regime for affinal parenthood. Judicial actors must therefore work with adjacent categories: affinity, third-party visitation, constitutional family protection and the child’s best interests.

The problem is not judicial interpretation as such, since every legal rule requires interpretation. The specific difficulty in the Peruvian case is that courts must interpret open and adjacent categories without a specific statutory framework governing post-divorce stepparent visitation. This increases the importance of judicial reasoning and the risk of inconsistent outcomes. A court could grant visitation when evidence shows stable caregiving, meaningful attachment and child-centered benefit. A court could also deny it where the bond is weak, where contact would intensify conflict or where the claim appears to serve the adult rather than the child.

## 3 Materials and methods

This study uses an exploratory qualitative socio-legal design. It combines analysis of formal legal materials with expert interviews in order to examine both law in books and law in action (Pound, 1910). The doctrinal component maps the constitutional, civil and child-protection categories that may support stepparent visitation in Peru. The empirical component examines how professionals involved in family disputes interpret the absence of an express post-divorce legal regime for affinal parenthood and how they reason about the child’s best interests, socio-affective bonds and evidentiary thresholds.

The study is not designed to produce statistical generalization. Its aim is analytic: to identify professional reasoning, interpretive patterns and points of tension in the legal recognition of stepparent contact after divorce. No inferential statistical analysis was conducted because the research question required qualitative interpretation of professional reasoning rather than hypothesis testing, prevalence estimation or statistical association.

The study was conducted in Ica, Peru, during the 2025 fieldwork period. The sample consisted of eleven adult professionals with direct legal, judicial, public-defense, prosecutorial or psychosocial experience relevant to family disputes, child protection, visitation and assessment of children’s welfare in family conflict: two family judges, four lawyers, two public defenders, one family prosecutor and two psychologists. The participant profile is summarized in Table 2. The sample was not intended to represent Peruvian judicial practice statistically. It was selected to obtain specialized professional reasoning from actors directly involved in the legal and psychosocial treatment of family disputes.

**Table 2:** Participant profile.

Code	Role	Field	Relevance
P1-P2	Family judges	Judicial branch	Judicial interpretation of family disputes
P3-P6	Lawyers	Legal sector	Family law reasoning on affinity and visitation
P7-P8	Public defenders	Public defense	Access to justice and child protection
P9	Family prosecutor	Public prosecution	Protection of children in family proceedings
P10-P11	Psychologists	Psychosocial assessment	Assessment of bonds and emotional welfare

Participants were selected through purposive convenience sampling. Inclusion criteria were professional knowledge relevant to family law, child protection, visitation disputes or psychosocial assessment; professional activity or expertise connected to the Peruvian legal system or Ica; voluntary willingness to answer the interview guide; and informed consent. Exclusion criteria were lack of relevant professional experience, participation as a litigant or directly interested party in a current stepparent-visitation case, inability to consent and incomplete responses.

Data were collected using a structured interview guide of ten open-ended questions organized around one general objective and four specific objectives. The guide asked participants to define visitation, affinity, family protection and socio-affective bonds; evaluate the viability of stepparent visitation after divorce; consider whether affinity after divorce is sufficient by itself; and explain the role of the best interests principle. Most expert interviews were conducted through written responses to a structured interview guide. Responses were then transcribed into a standardized analytical matrix.

All responses were anonymized before analysis. Participants were assigned codes P1 to P11. Names, institutional identifiers, signatures and other identifying information were removed. The anonymized response matrix and codebook were stored separately from consent forms and identifiable documents. Direct quotations are translated excerpts from Spanish interview materials. The translation was reviewed by the research team, and only minimal linguistic editing was made to preserve meaning, improve readability and avoid disclosure.

### 3.1 Analytical strategy, coding and triangulation

The interview data were analyzed through directed thematic analysis combined with socio-legal analysis. The analysis proceeded through familiarization with the interview material, first-cycle coding, development of a preliminary codebook, comparison of coded segments, refinement of themes and integration of themes with the legal framework (Braun and Clarke, 2006; Nowell et al., 2017).

The coding process followed four operational stages. First, each response was entered into a standardized matrix organized by participant code, professional role, interview question and initial analytical memo. Second, two coders independently assigned deductive and inductive codes. Deductive codes were derived from the theoretical framework, including law in books/law in action, socio-legal recognition, affinal parenthood, legal parenthood, social parenthood, functional parenthood, de facto caregiving, the best interests of the child, socio-affective kinship, child contact and judicial discretion. Inductive codes emerged from responses, including insufficiency of formal affinity alone, need for psychological assessment, prior caregiving, non-displacement of biological parents, emotional continuity and risk of unequal decisions.

Third, coded segments were compared to identify convergence, divergence and ambiguous classifications. Fourth, disagreements were discussed with a third team member until a consensual thematic category was assigned. Disagreements were not treated only as errors; they were used to identify ambiguous categories, especially where participants differed on whether formal affinity was sufficient or psychosocial proof of a meaningful bond was required.

Triangulation was used as a concrete analytic comparison, not as a generic claim of validation. The study compared interview responses, statutory provisions, constitutional jurisprudence, Peruvian doctrine and international socio-legal literature. This comparison was used to identify convergences, divergences and tensions between formal legal categories and professional reasoning. It was not used to claim statistical or universal validity. The research team also considered its disciplinary position during coding: legal researchers focused on doctrinal coherence, whereas the psychosocial reviewer examined whether the interpretation of attachment, emotional continuity and risk remained faithful to the interview material.

Because this is an exploratory expert interview study, sample adequacy was assessed through information power rather than numerical representativeness (Malterud et al., 2016). The study does not claim saturation in a strong universal sense. It claims only that the interviews were sufficient to identify recurring and theoretically relevant patterns within the bounded expert field examined.

## 4 Results

The analysis identified six interrelated themes. Formal affinity operated as a procedural gateway, but not as a complete answer. The best-interests principle was treated as a structured judicial inquiry. Socio-affective bonds functioned as an evidentiary threshold. Participants emphasized the non-displacement of biological or legal parents. Psychosocial evidence was considered necessary to evaluate attachment and risk. Finally, participants identified statutory silence as a source of judicial uncertainty. The six themes and their socio-legal meaning are summarized in Table 3.

**Table 3:** Themes and socio-legal meaning.

Theme	Supporting material	Socio-legal meaning
Formal affinity as gateway	Participants linked Article 237 with persistence of affinity after divorce.	Affinity makes the claim legally intelligible but not automatically valid.
Best interests as structured inquiry	Participants treated welfare, stability and development as decisive.	The standard connects statutory silence with lived family relationships when supported by evidence.
Socio-affective bond as threshold	Participants emphasized care, cohabitation and prior parental functions.	Recognition depends on the relationship lived by the child.
Non-displacement of legal parents	Participants distinguished visitation from custody and parental authority.	Contact must be complementary and child-centered.
Interdisciplinary evidence	Psychosocial actors stressed attachment and professional assessment.	Psychological evidence structures the best-interests inquiry.
Judicial uncertainty	Participants noted absence of specific regulation.	Flexibility may protect children but also produce uneven decisions.

Participants generally recognized that the persistence of affinity after divorce can provide an initial legal basis for discussing stepparent visitation. However, they did not treat affinity as sufficient by itself. A public defender explained: “Affinity can support the request, but it should not be understood as automatic. The judge must verify whether there was a real bond with the child and whether contact would be positive for the child’s development” (P7, public defender; translated from Spanish). A second public defender added: “The existence of affinity is a legal starting point, but it must be accompanied by evidence of affection, coexistence and a parental role. Otherwise, the claim would only be formal” (P8, public defender; translated from Spanish).

The best interests principle was the strongest point of convergence across professional groups. Participants described it as the criterion that allows judges to move beyond a formal assessment and evaluate whether continued contact protects emotional stability, identity and development. A family prosecutor emphasized: “The best interests of the child require an individualized evaluation. It is not enough to invoke the principle; it must be shown that the visit preserves the child’s welfare and does not expose the child to conflict or harm” (P9, family prosecutor; translated from Spanish).

Participants repeatedly emphasized that the decisive issue is not the label of padrastro, madrastra, padre afín or madre afín, but the quality of the relationship that existed before the marital breakdown. One psychologist summarized: “The bond cannot be reduced to the legal label. It must be observed in daily life: who cared for the child, who accompanied the child, and whether the child identifies that person as emotionally significant” (P11, psychologist; translated from Spanish).

Another recurring concern was that stepparent visitation should not displace the rights and duties of biological or legal parents. Participants distinguished child contact from custody, parental authority, adoption and legal parenthood. A public defender stated: “The stepparent’s contact should be complementary. It should not replace the father or mother, nor interfere with parental authority. The question is whether maintaining contact helps the child” (P8, public defender; translated from Spanish).

Psychologists and child-protection professionals emphasized that socio-affective bonds cannot be fully assessed through legal documents alone. One psychologist noted: “A psychological evaluation is useful because it can identify whether the relationship is secure, whether the child wants or rejects contact, and whether visitation would produce stability or distress” (P10, psychologist; translated from Spanish). These responses suggest that psychosocial evidence can help operationalize the best-interests standard.

Finally, participants identified the absence of express regulation as a source of uncertainty. Several interviewees considered that courts have legal tools to admit and evaluate a stepparent visitation claim, but also recognized that lack of statutory criteria may generate inconsistent outcomes. The interviews therefore converged around a structured judicial inquiry: affinity may justify standing, socio-affective evidence should determine seriousness, psychosocial assessment can support the best-interests evaluation and contact should be granted only when it protects the child without displacing legal parents.

## 5 Discussion

The findings show that the legal recognition of stepparent visitation in Peru cannot be understood as a simple doctrinal question about the scope of Article 237 of the Civil Code or Article 90 of the Code of Children and Adolescents. Those provisions matter because they make affinity and extended visitation legally intelligible. However, the interviews show that professionals do not treat formal affinity as a complete answer. They understand it as a gateway that must be completed by evidence of social or functional parenthood, caregiving, emotional continuity and the child’s best interests (Bazán, 2022; Mason et al., 2002; Varsi, 2020).

The first contribution of the study is to show how Peruvian professionals distinguish between affinal status and socio-affective parenthood. Participants did not support automatic legal parenthood for stepparents, but they did support judicial evaluation of meaningful child-stepparent contact where the adult had performed a stable parental role. This middle position avoids two extremes: denying any legal relevance to stepparents after divorce because they are not legal parents, and treating stepparenthood as equivalent to full legal parenthood (Malia, 2005; Huntington et al., 2023; Lind, 2024).

The argument does not claim that Peru is unique because it uses the best interests of the child. The Peruvian contribution lies in the combination of constitutional recognition of blended families, statutory silence regarding post-divorce stepparenthood and open legal categories such as affinity and third-party visitation (Meza et al., 2019; Shinno, 2023; Villa and Hurtado, 2022). This combination makes Peru a useful case for studying how legal actors translate open standards into concrete reasoning when the statute does not provide a specific regime.

Comparative family law also requires caution. It is not accurate to claim that all legal systems privilege form over function. Many systems continue to organize parenthood primarily through formal categories such as filiation, adoption, marriage and parental responsibility. However, several jurisdictions have developed functional or fact-sensitive mechanisms for recognizing cohabitation, de facto caregiving, social parenthood, psychological parenthood or contact rights in particular circumstances (Harding and Pawliczak, 2025; Lima, 2019; Scherpe, 2005). The Peruvian case should therefore be situated within a broader movement from exclusive reliance on status-based categories toward more functional, child-centered forms of legal recognition.

The findings also support a structured best-interests model. Courts should ask whether the stepparent performed caregiving functions, whether the child developed a meaningful bond, whether the child’s identity and stability would be affected by interruption of contact, whether the child wishes to continue or reject contact according to age and maturity, whether the relationship exposes the child to conflict or harm and whether visitation would interfere with legal parents (Archard and Skivenes, 2009; Brown and Wade, 2023; König et al., 2024). This model does not eliminate judicial discretion, but it makes discretion reasoned and reviewable.

The inclusion of public defenders, a family prosecutor and psychologists shows that the evidentiary problem is not only legal. Courts should not infer socio-affective kinship from marriage alone or rely only on the stepparent’s assertion that a bond exists. Psychosocial assessment can help identify whether contact would provide stability or create distress, whether the child recognizes the stepparent as significant and whether continued visitation would intensify adult conflict.

The study has limitations. The sample is small and limited to expert participants connected to the Peruvian legal and psychosocial field, with particular emphasis on Ica. It does not include children, biological parents, stepparents, other members of blended families or full case-file analysis. The results should therefore be read as an exploratory account of professional reasoning rather than as a statistically generalizable description of Peruvian judicial practice. Future research should incorporate child-sensitive methodologies, interviews with affected families and comparative case-file analysis (Archard and Skivenes, 2009; Smart, 2006).

## 6 Conclusion

This article examined how Peruvian judicial, legal, prosecutorial, public-defense and psychosocial professionals interpret the possibility of granting visitation rights to stepparents after divorce in blended families. The findings indicate that formal affinity can provide a legal entry point, but it does not automatically justify visitation. The decisive factor is whether the relationship between the child and the stepparent became socio-affectively significant and whether continued contact serves the child’s best interests without displacing the rights and duties of biological or legal parents.

The central socio-legal insight is that the best interests principle operates as both a bridge and a risk. It is a bridge because it allows courts to consider lived family relationships that the statute does not expressly regulate. It is a risk because, without clear criteria, it can lead to uneven judicial outcomes. A structured approach should therefore require evidence of caregiving, cohabitation, emotional attachment, child identity, absence of harm and psychosocial assessment when necessary.

The Peruvian case contributes to wider debates about social parenthood, functional parenthood, socio-affective kinship, law in action and judicial discretion in family law (Huntington et al., 2023; Pound, 1910). Legal recognition of affinal parenthood should not be understood as a replacement for biological or legal parenthood, but as a carefully limited child-centered mechanism to preserve meaningful relationships where continuity is demonstrably beneficial for the child.

## Data Availability

The original contributions presented in the study are included in the article/supplementary material, further inquiries can be directed to the corresponding author.
